# Exploring mothers' and grandmothers' perceptions of animal-source complementary foods in the diets of young children in The Gambia: A qualitative study

**DOI:** 10.12688/wellcomeopenres.23090.2

**Published:** 2025-07-23

**Authors:** Emily Dawson, Ahmed Futa, Maaike Klappe, Carla Cerami, Hilary Davies-Kershaw

**Affiliations:** 1 London School of Hygiene & Tropical Medicine, London, England, WC1E 7HT, UK; 2 Medical Research Council Unit The Gambia at the London School of Hygiene and Tropical Medicine, Fajara, Banjul, N/A, The Gambia

**Keywords:** Complementary feeding practices; Complementary foods; Animal source foods; Qualitative analysis; The Gambia; mothers; grandmothers

## Abstract

**Background:**

In The Gambia, many children consume diets that are lacking in nutrients that are essential for their growth and development. This study aims to explore Gambian mothers' and grandmothers' perceptions around animal source foods (meat, eggs, milk etc.) in order to inform future interventions focused on improving child feeding practices.

**Methods:**

In July and August 2023, nine semi – structured focus group discussions were conducted with mothers and grandmothers in two settings in The Gambia. A purposeful sample of participants were recruited with the support of each local Medical Research Council clinic. Data were analysed using the framework method and thematic analysis. Participants included 19 mothers and 12 grandmothers in a semi-rural area and 12 mothers and 12 grandmothers in a rural area, caring for children 6–24 and grandmothers were in separate groups.

**Results:**

Knowledge gaps were identified in both settings regarding aspects of complementary feeding, such as the appropriate timing and methods for introducing animal source foods to children’s diets. Differences were noted in the availability and dissemination of infant and young child feeding information in the two settings, emphasising the crucial role of contextual factors in shaping future programmes. The accessibility and affordability of animal source foods, was found to be a key determinant of their inclusion into children’s diets. A reliance on commercially available complementary foods was common in both settings.

**Conclusion:**

This study reveals disparities between current complementary feeding practices and guidelines in this setting. It also underscores context-specific barriers caregivers encounter in offering diverse complementary foods, including animal source foods. A high reliance on commercially available complementary foods was also uncovered, particularly in Keneba. Further research is recommended to aid the development of context- specific interventions.

## Plain language summary

In The Gambia, many children consume diets that are lacking in nutrients that are essential for their growth and development. This study aims to explore child feeding practices, including perceptions around animal source foods (meat, eggs, milk etc.) in order to inform future interventions focused on improving child feeding practices. In July and August 2023, investigators held focus groups with mothers and grandmothers in both Keneba, a rural village and in Brikama, an urban town, in The Gambia.

This study finds discrepancies between recommended feeding guidelines and actual practices, influenced by various local factors. Understanding these influences is an essential first step in working to improve complementary feeding practices in The Gambia. This study also looks at the relationship between the Medical Research Council, The Gambia (MRCG) and the community in both settings. This provides insight into the appropriateness of the use of the MRCG as a setting for an intervention. The insights from this research are able to guide future efforts to improve child feeding practices in The Gambia. The main limitation to the study was the lead investigators lack of fluency in the local language, however support from local researchers aided the lead researcher’s comprehension.

## Introduction

A child’s first 1000 days of life, the period from conception to the second birthday, is a critical time to receive the nutrients that support their growth and development ^1^. Current World Health Organisation (WHO) recommendations state that after a six month period of exclusive breastfeeding, nutrient-dense, high-quality complementary foods should be introduced into children’s diets alongside breastmilk for their first two years to ensure that their nutrient requirements are met ^2^. Complementary foods encompass all solid or liquid foods introduced to children, excluding breast milk or commercial milk formula, while complementary feeding practices refer to the food consistency, content, and meal frequency alongside preparation and storage techniques ^3^. In low resource settings, such as The Gambia, appropriate complementary foods and feeding practices can facilitate long-term health and productivity, thereby aiding in breaking intergenerational cycles of poverty ^4^.

Whilst breastfeeding rates in The Gambia have been reported to be improving, with recent data showing that 98% of children are currently being breastfed ^5^, the quality of complementary foods remains poor. Currently, consumption of animal source foods, which provide protein and essential micronutrients including iron, is limited ^6^. Instead, children’s diets are high in carbohydrates, mostly comprising of cereals such as millet and corn ^7^. Whilst these cereals provide important carbohydrates, the low levels of micronutrients they do contain such as iron, calcium and zinc are often bound by phytate, an anti-nutrient which hinders absorption ^8– 10^. Therefore, cereals alone are unlikely to provide children in this setting with all essential nutrients ^11, 12^. Studies found that these suboptimal feeding practices are contributing to concerning rates of micronutrient deficiencies in children in The Gambia ^7^, where 59% of children aged six to 59 months suffer from iron deficiency, with 38% experiencing iron deficiency anemia ^13^.

The WHO recommends early introduction of animal source foods such as milk, eggs, meat, and fish into children's diets as sources of complete protein and as an effective way to improve the nutrient density of complementary foods ^2^. Their addition to children’s diets has been shown to decrease anaemia ^14^, aid cognitive development ^15^ and induce better tissue growth ^16^. In contrast to plant sources, animal sources provide haem iron, a highly bioavailable form that is easily absorbed by the body ^17^. Even the consumption of relatively small amounts can contribute substantially to dietary adequacy ^18^. In addition, providing children with a variety of animal source foods can contribute to dietary diversity, which acts as a proxy measure for dietary adequacy and micronutrient sufficiency ^19^.

Many households in Sub-Saharan Africa have low consumption of animal source foods due to factors such as the availability, accessibility and affordability of these foods ^20^. One significant factor is the insufficient availability of these foods, often linked to poor infrastructure ^21^ and low productivity of livestock ^22^. In addition, social and cultural norms can influence the consumption of animal source foods among children ^23^. Often when animal source foods are available, social patterns with household food allocation favour older males, thus leading to lower consumption in children ^20, 24, 25^. It is evident that determinants are complex and context specific. Consequently, addressing them poses considerable challenges, and effective methods to overcome them are currently limited ^18^.

Qualitative studies on complementary feeding are available for many parts of Sub-Saharan Africa ^26– 30^, however are lacking for The Gambia. In addition, data on the perceptions of animal source foods is scarce. Given the low consumption of animal source foods in this age-group and the known benefits on infant health, better knowledge and understanding of care givers perceptions of these foods is important for the development of culturally suitable and context-specific complementary feeding interventions. As the main caregivers, mothers play an important role in children’s feeding in The Gambia ^30^. Similarly, grandmothers, often esteemed as authoritative figures within families and communities, also play a significant role ^31^. Thus, it is crucial to take into account the knowledge and beliefs of both mothers and grandmothers.

This qualitative study used focus group discussions (FGD) with Gambian mothers and grandmothers to (i) explore Gambian caregivers’ perceptions of animal-source complementary foods and (ii) explore Gambian caregivers’ current complementary feeding practises.

## Methods

### Participants

Study participants were recruited from two locations in The Gambia: Brikama, a semi-urban town and Keneba, a rural village, due to their proximity to a Medical Research Council, The Gambia (MRCG) and thus access to data for recruitment and trained field workers. It also provided an opportunity to compare and contrast between an urban and a rural setting. Nine FGDs were carried out, five in Brikama (comprised of 19 mothers and 12 grandmothers) and four in Keneba (comprised of 12 mothers and 12 grandmothers). To encourage an environment conducive to open and unrestricted dialogue, mothers and grandmothers were enrolled into separate FGDs.

With the support of the MRCG, a purposeful sample of participants caring for at least one child aged six to 24 months was identified in both settings. In Keneba, the West Kiang Demographic Health Surveillance System (DHS) was utilised ^32^. In Brikama, participants were approached from communities around the health centre, due to lack of a DHS. The investigators and field workers, visited potential participants homes, explaining the study, the researchers interests and enrolling willing participants. There were no exclusion criteria for ages of participants or for the number of children they cared for, however all participants cared for or had cared for at least one child. This aligned with the method's goal of capturing diverse knowledge and beliefs ^33^.

### Data collection

FGDs were held at the MRCG sites in both settings. In Keneba they were facilitated by the lead investigator, and another qualitative researcher, who then facilitated the FGDs in Brikama. Both researchers were qualitatively trained students completing their MSc research project. Both FGDs were supported by field workers from the local area. A semi- structured topic guide was used during the FGDs, the main themes were: (i) exploring complementary feeding practises, (ii) perceptions of animal source complementary foods and (iii) access to appropriate complementary foods. The guide was developed with guidance from MRCG staff, to ensure that questions were culturally relevant and appropriate, then piloted with a small group of mothers.

Prior to beginning the FGDs, training was carried out with the field workers to ensure that the study aims were understood. This solidified the roles of the facilitator and the field worker and ensured quality of data collection. During FGDs, questions were asked in English by the facilitator, and translated into Mandinka by the field worker. The participants responded in Mandinka which was translated back into English to the facilitator. Translation was undertaken by the field workers from each corresponding MRCG site. The FGD’s were recorded on two devices and field notes were taken, which were then used for data triangulation and further translation. FGD’s lasted between 45 to 60 minutes. Please see the Extended Dataset.

### Data analysis

The framework method was used for analysis, as outlined by Gale *et al*. ^34^. This method involves transcription, familiarisation and coding, followed by the development and application of a framework, charting the data into a matrix before interpreting. This systematic approach facilitated structured data management, enabling comparison across the participant groups and the identification of key themes and relationships. This was considered an appropriate method to compare and contrast the data. From the recorded FGDs the English translation was transcribed verbatim due to Mandinka being only a spoken language. Transcripts were then re-read and reflective notes were taken to identify any thoughts or impressions of the data. Initial codes, capturing main themes, were applied through open coding using NVivo, alternatively this could’ve been carried out using Excel. Emerging codes led to re-reading and recoding prior transcripts. For the Keneba data, these steps were carried out by the lead investigator and by the second investigator in Brikama. The transcription and initial coding from both data sets was then read by the lead investigator and the data were discussed.

**Figure 1.**
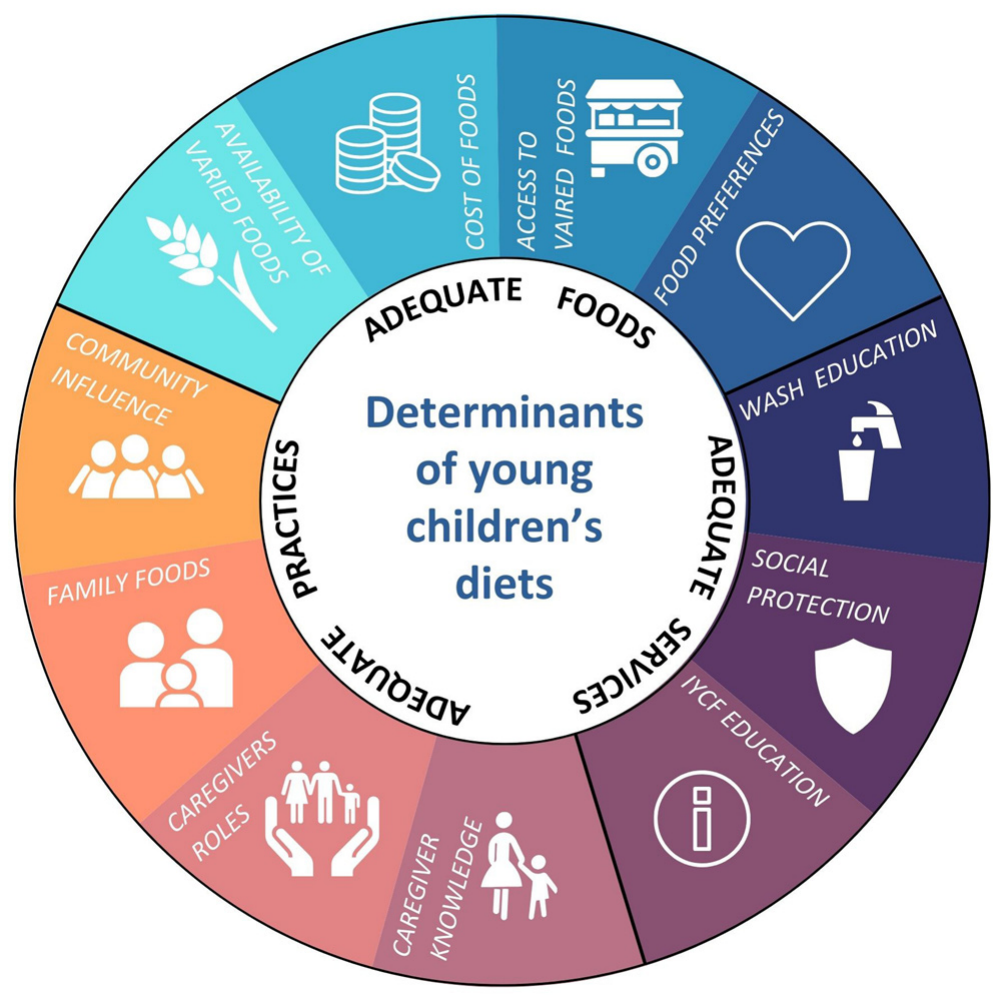
Framework adapted from UNICEF’s determinants of young children’s diets'.

The lead investigator then compared and combined the codes from both data sets to form subthemes which aligned with the topic guide themes. Subthemes were continually reviewed and organised to create an analytical framework that addressed the overall research aims and questions. This was then applied to all of the transcripts. This allowed for a comprehensive analysis and interpretation of the data, ensuring that crucial insights and findings were effectively identified and explored. A framework from the United Nations Children’s Fund’s (UNICEF) *Determinants and drivers of young children’s diets during the complementary period* ^12^ was used to inform a framework matrix. The framework identified adequate foods, practices, and services as determinants of good diets for young children, which are influenced by context-specific factors known as drivers ^12^. These were used by the lead investigator to create a framework which the subthemes and codes were charted onto ( Figure 1.) This aided the organisation and interpretation of the data by exploring the foods, practices and services that the mothers and grandmothers are using to support their children’s complementary feeding. Social protection and WASH education, were not identified in the data so are not discussed.

### Ethical approval

This study was conducted according to the guidelines laid down in the Declaration of Helsinki and all procedures involving research study participants were approved by the The MRC Scientific Coordinating Committee ((LEO28788, approved on 24 May 2023), The Gambia Ethics Committee (LEO28788, approved on 19 June 2023) and the LSHTM Ethics Committee (LEO28788, approved on 28 June 2023),

## Discussion of results

### Study participants

The mean age of mothers and grandmothers was 33 and 60 respectively. The majority of mothers were housewives. The ages of the children in the mothers’ care were evenly spread across the three age groups, however grandmothers more commonly cared for children that were 12 – 24 months (see Table 1).

**Table 1.** Characteristics of mothers and grandmothers that participated in focus groups.

	Mothers			Grandmothers		
Characteristics	Mean	n	%	Mean	n	%
Age (years)	32.7	28		59.8	24	
**Occupation**						
Housewife		14	50		9	38
Business woman / vendor		2	7		3	12
Cleaner		2	7		0	0
Teacher		3	11		0	0
Market vendor		7	25		2	8
Unknown		0	0		12	50
**Number of children**						
1 to 2		8	29		8	33
3 to 4		7	25		6	25
5 to 8		12	43		9	38
8+		0	0		1	4
unknown		1	4		0	0
**Age of youngest child**						
Under 6 months		6	21		3	13
6 – 11 months		8	29		6	25
12 – 24 months		10	36		14	58
Unknown		4	14		1	4

Ten subthemes, identified in the data from grouping codes, were charted onto the framework based on UNICEF’s determinants of young children’s diets ^12^ as a way of organising and interpreting the data ( Figure 1). These came under UNICEF’s three overarching themes, which has structured the discussion of the results.

### Theme 1: Adequate practices

***Caregiver knowledge***

Research shows that caregivers play a pivotal role in determining the dietary patterns of children ^12^. This study explored aspects of caregivers’ knowledge of IYCF (Infant and Young Child Feeding) including exclusive breastfeeding and complementary feeding practices. In both Brikama and Keneba, mothers and grandmothers were able to list various animal and plant source foods that are good for children. Fruits were commonly mentioned and included banana, apple, orange and mango. Animal source foods included fish, yoghurt, milk and meat. Generally, their awareness of foods suitable for children aligned with the WHO recommendations for complementary feeding, which define the eight key food groups for children as: 1) breast milk; 2) flesh foods 3) dairy 4) eggs; 5) legumes and nuts; 6) vitamin-A rich fruits and vegetables; 7) other fruits and vegetables; and 8) grains, roots, and tubers ^2^.

Similarly, mothers and grandmothers in both settings agreed that animal source foods are ‘ *good for their (child’s) body’* (K1G5), *‘gives the child strength’* (B5G4) and helps them to avoid *‘small sicknesses; fever, diarrhoea and vomiting.’* (K1G2) However, this was as much detail as they could provide, suggesting that the advantages of children consuming animal source foods were not adequately understood. Studies in Sub-Saharan Africa have shown that this limited understanding leads to lower consumption of animal source foods among children ^35, 36^. Caregivers who have a more comprehensive understanding of child nutrition and its importance for overall growth and development are more likely to provide children with high-quality diets ^36^.

Caregivers’ knowledge about the timely introduction of complementary food has also been shown to be a primary factor of facilitating good practises ^27^. In this study, the reported age that complementary foods were introduced varied. Mothers consistently stated the WHO recommended six months, while grandmothers more frequently said younger.

*“At three months, I start to introduce solid foods like porridge.”* (B5G6)

*“(The child) cannot eat until three months. Then that child can eat. Then she prepares food for her.”* (K1G1)

Grandmothers appeared to be less familiar with the current recommendations which changed in 2003, from three to six months following updated research that showed benefits to longer periods of exclusive breastfeeding ^37^. This shift may have caused confusion among grandmothers, resulting in the range of responses.

Reasons behind the age of introducing complementary foods was also explored. The most prominent belief across both settings was the need for children to have teeth prior to introducing animal source foods, such as meat and fish, into their diets.

*“If it (the child) does not have teeth (they) cannot eat. But immediately if he have teeth the child will take it.” (K2M3)*

*“Without that (teeth) you cannot take it. Yeah, because you have to chew it before it digests.” (K1G1)*

*“When the child starts to grow teeth and the teeth are strong and can chew meat then we introduce meat.” (B5G4)*

However, guidelines report that if appropriately prepared, meat and fish are able to be included into diets from six months, at which point not all children have developed teeth as this usually happens between six and 12 months ^2^.

In Brikama, food taboos were brought up by participants. Some mothers and grandmothers mentioned concerns about negative behaviour and impaired brain development, as reasons for avoiding certain animal source foods for children.

*“We avoid giving meat because if you go for a visit, the child will be tempering (angry) with meat on the table.”* (B3M2)

*“If you introduce eggs to the child, sometimes the child may not speak and may become dumb.”* (B3M4)

However, others stated they *‘don’t believe in these taboos.’* (B5G1)

*“I don’t believe in the taboo that pregnant women should not eat eggs. Even the day I went into labour I ate eggs.”* (B4M3)

***“For the kids, they can eat banana and eggs. It doesn't affect them.*” *(B5G4)***

***“For the kids, they can eat banana and eggs. It doesn't affect them.” (B5G4)***

Similar beliefs about animal sourced foods have been recorded in other regions of The Gambia and may be contributing to reduced animal source food consumption ^38^. This is particularly harmful given the high levels of anaemia ^39^, which can be reduced by increased consumption of these foods ^14^. It is therefore important that beliefs surrounding complementary feeding practices are considered during interventions.

Commercially available complementary foods (CACF) were brought up by participants in both settings, with mixed opinions on whether they were good for children. In Keneba, there seemed to be a general consensus that they had benefits.

*“It (CACF) is good for the baby and the baby will always have good weight and good health.”* (K3M6)

*“Sometimes it (CACF) is good for the children and sometimes some is not good for them.”* (K1G2)

In Brikama, CACF was spoken about more negatively, with concerns about the safety of the products.

*“I believe that the local cereals give more health to the child than the cerelac… the shop food has been prepared by somebody and I don’t know the content of it. That’s why I trust the homegrown foods.”* (B3M6)

*“These tin foods are good for the child if they are in proper care condition. But when they get expired it’s not safe to give to the child.” (B5G1)*

The origin of these differing beliefs is unclear due to a lack of research on this topic. It is possible that the limited availability of diverse foods in Keneba (see Theme 3) has led mothers and grandmothers to rely more heavily on CACF compared to those in Brikama. As a result, they may be more inclined to view CACF more positively, believing it to be beneficial for their children’s weight and health.

The study revealed significant disparities in caregivers’ knowledge about IYCF, particularly in complementary feeding. Notably, grandmothers were less familiar with the current recommendations on the age at which to introduce complementary foods. These findings suggest that education on complementary feeding has been either sparse or ineffective, and that context-specific beliefs should be addressed to improve caregiver knowledge.

***Community influence***

Communities influence feeding practices in many ways. In Sub-Saharan Africa sharing knowledge within and between families is a commonly used channel of IYCF education ^29, 40^. To explore this, mothers and grandmothers were asked about how they acquire IYCF education that influences their feeding practices. In Keneba, there seemed to be a well-established culture of sharing knowledge, including IYCF, between families and within the community.

*“Here also in the village, they have they have selected people who are responsible for that (IYCF)…they teach them.”* (K3M5)

*“When your child is sick, the people … they show them how to prepare (food). Maybe in the village they saw some so that they can help each other.* (K2M5)

In addition, grandmothers spoke of the Alkalo (community leader) appointing people from the village to share IYCF education.

*“Village representatives do the announcement at the bantaba (communal area), clinic or at the mosque.”* (K2G5)

In Brikama, members of the community such as *‘friends and colleagues’* (B4M2) were occasionally mentioned as providers of knowledge.

In Keneba, the collaboration between public health initiatives and influential community members emerged as crucial for the success of such dissemination efforts. However, the powerful influence of the community on complementary feeding practices which was seen in Keneba was not as apparent in Brikama. This may be due to Brikama having a larger population and being more urban than Keneba, where members of the community are more familiar with each other. These findings highlight the need for context-specific strategies to effectively disseminate IYCF-related information.

***Family foods***

Children’s diets are frequently influenced by the dietary habits of their families. Consequently, participants were asked about the foods consumed within their households.

In both settings, participants spoke of growing many fruits and vegetables that are available for the family to eat.

*“Women here they have a big garden … so they always go there and prepare foods for the family like vegetables, garden eggs, pepper, green beans and onion. Then they will sell some and they eat some and when they sell, they also support the family.”* (K1G2)

*“I also have a small garden in the compound where I grow okra, sorrel, and some small vegetables that I can cook for the family.”* (B5G4)

In both settings, mothers and grandmothers spoke of eating purchased animal source foods (see ‘availability of diverse foods’). In Brikama, mothers mentioned owning livestock, particularly during the recent Islamic festival Tobaski. This was not discussed in Keneba, however, it was apparent that there was livestock in the village.

*“We have chickens, sheep and goat at home” (B4M3)*

*“We also rear some animals, but they are in the village. I don't keep them with me but anytime I need any I can get them.” (B1M6)*

*“We have a lot of animals like sheep at home. We usually eat the sheep during Tobaski” - (B1M2)*

Whilst they often mentioned giving the produce they grow to their families, many of the foods they reported growing or rearing were not mentioned when asked what foods were fed to children under 24 months. This suggests a discrepancy between the available family foods and those provided to young children, indicating potential areas for intervention to improve the inclusion of animal-source complementary foods in children's diets.

***Caregiver roles***

Work and household responsibilities limit caregivers' time and can hinder their ability to prepare and feed children nutritious foods as frequently as recommended ^12^. The roles of caregivers and the effect that these roles have on complementary feeding were explored in this study. Participants listed numerous paid and unpaid responsibilities that they carried out around child care, with differences being seen across the two settings.

In Keneba, participants noted that the current wet season led to a sense of busyness. Mothers spoke of having busy morning routines.

*“During this time (rainy season), everyone is busy. Everyone is busy.”* (K2G2)

*“Sweeping, laundry, going to the garden from the garden go to the market and cook the lunch. After cooking lunch then she go back to the garden again until around 6pm.”* (K2M1)

Their engagements encompassed tasks they perceived as integral to their roles as women.

*“Because they are the woman and they are always with the family. They take care of the family more than the men.”* (K2M1)

In Brikama, mothers were more likely to work in a business alongside their husbands to provide for the family.

*“I am working, and my husband is also working. At the end of the month my husband provides money to buy food. It is his responsibility. But sometimes when my husband doesn't have money, I buy food for the child.” (B1M6)*

In both settings, participants spoke of their many responsibilities as a woman, shedding light on gender-based roles in this setting and their implications for caregiving practices. These findings emphasise the need for public health programmes to acknowledge and accommodate caregivers' time constraints in promoting optimal complementary feeding practices. Efforts should consider flexible approaches that support caregivers in balancing their responsibilities while ensuring adequate nutrition for young children.

### Theme 2: Adequate services

***IYCF education***

The provision of IYCF education services in both settings was discussed. Knowledge of current breastfeeding guidelines was observed amongst all participants across both settings. Most mothers and grandmothers demonstrated a strong understanding of exclusive breastfeeding and were able to refer to the current WHO guidelines ^3^.

*“It's breastfeeding without giving the child water or food until she's six months.”* (K3M4)

“ *It’s the only the breastfeeding. No food except the medication or supplement…until she is six months.”* (K2G4)

*“The breastmilk contains everything, all the nutrients for the child so breastfeeding the child for six months without water or liquid.” (B4M4)*

Thus, this study indicates that breastfeeding education services are prevalent and effective in both contexts. However, as previously mentioned, knowledge on some aspects of complementary feeding recommendations appears to be less robust, suggesting that services providing education focussed on complementary feeding is less so.

In Keneba, when asked where they acquired knowledge on exclusive breastfeeding, available services were mostly spoken about with regard to the health and nutrition services provided by the government and the MRCG clinic.

*“The government always comes here. So, they come to here, and teach them how to breastfeed.”* (K1G6)

*“Immediately after birth, the nurses will explain it to you that you should not give anything to the child until she's six months.” (K3M4)*

The MRCG clinic provides free health care in Keneba, which includes an outpatient service with a concentration on maternal and child health ^41^. In addition, as part of the Gambian government's National Development Plan ^42^, communities should receive IYCF education via visits from public health nurses. Although details of these visits are sparse, these findings indicate that education on exclusive breastfeeding has improved knowledge in Keneba.

The now closed MRCG supplement centre was also mentioned as a service that supported mothers with complementary feeding. Where they were able to learn how to make nutrient-rich complementary foods from ‘ *groundnut paste, millet, oil and fish’* (K1G2).

*“Now the supplement centre is not there. Some years back, it was helping them very much.”* (K2M2)

In Keneba, the MRCG Nutrition Supplementation Centre was an integral part of the nutrition services available from when it was established in 1976 to when it closed in recent years due to lack of funding ^30^. Studies found the centre to effectively carry out interventions including providing food supplements, educating caregivers on child nutrition practices, basic hygiene, and food preparation methods through intervention programs ^30, 43– 45^. This, along with the work of the wider MRCG clinic has exposed the community to a substantial amount of child health and nutrition knowledge and awareness ^30^. The centre’s success is evident in this study, where participants shared their learning experiences from it.

However, in Brikama, mothers and grandmothers referenced ‘ *elders’* (B3M2) or other family members such as ‘ *husband’s sisters’* (B3M6) or *‘aunts’* (B3M4) as their main source of IYCF education.

*“I have never gotten any education outside, like in the community or health centre. I have learned everything from my mum.”* (B2G6)

When IYCF education services were mentioned, they referred to the government-run Immunisation Programme (EPI) and *‘antenatal clinic’* (B3M4). They also mentioned the Christian Child Fund (CCF), a Non-Government Organisation (NGO) and programmes on the radio.

*“When I go (to the) EPI clinic, they give us health education. about how to care for the child.”* (B1M6)

*“(CCF) also teach us how to prepare good food for the child also teaches us about exclusive breastfeeding.”* (B4M3)

*"I obtain information from the radio. Sometimes on the radio, they host discussions on how to prepare healthy baby food."*(B3M2)

In Brikama, external IYCF education was limited, with participants primarily relying on familial knowledge. The government-run EPI clinic was cited as the primary source of external IYCF education. This underscores the lesser exposure to comprehensive IYCF education among participants in Brikama compared to Keneba and the difference in where it is acquired. Despite this difference, it was clear that breastfeeding education was being provided in both settings, providing an opportune time to also deliver education on other aspects of IYCF including complementary feeding.

### Theme 3: Adequate foods

***Availability of diverse foods***

In Sub-Saharan Africa, poor availability of diverse foods affects complementary feeding practices ^27, 46^. This was reflected in the findings from Keneba, where lack of availability of meat was continuously identified as a barrier to consumption. Fish was said to be more readily available than meat and therefore more commonly consumed.

*“If there is no meat, every day fish. Fish is very easy. That one. Now it is available. Not always, but it's better than meat. They eat that more than meat.”* (K2M1)

*“Fish is very easy to have in the Gambia. But meat it's not easy. Sometimes two to three months before they will have meat to eat.”* (K1G6)

In Brikama, both mothers and grandmothers often mentioned animal source foods being readily available in *‘markets’* as well as rearing their own livestock. This contrasts with Keneba, where such availability is limited. The difference is likely due to Brikama's status as a well-connected urban area compared to the more isolated rural setting of Keneba.

The Gambia has a seasonal agricultural system that revolves around a rainy season from July to November and a dry season from November to May. Therefore, production of food, including those of animal source, is heavily dependent on the weather, and it is common for households to bridge a food deficit during the rainy season ^21^. This was reflected in both settings, where mothers and grandmothers spoke of the effects of seasonality on food availability and the difficulties that this causes them.

*“During the mango season, the child likes that. Even orange season. The same thing. During the rainy season we have yoghurt here. But during the dry season we don't have it.”* (K1G1)

*“In the rainy season, it’s tough to get food even in the market. In the dry season there is a lot of fresh food in the market.”* (B3M6)

Based on their accounts, it became evident that the availability of foods, particularly in Keneba, significantly impacted the dietary patterns of children.

***Cost of foods***

The Gambia is currently facing increases in food prices due to its heavy dependence on imports, rendering it vulnerable to global inflation ^21^. As a consequence, the affordability of numerous food products has been significantly impacted, leading to a considerable burden on household welfare, with rural areas experiencing the most severe effects ^47^. In both settings, meat was considered expensive which influenced the low and infrequent consumption by families.

*“Here meat is very expensive. So sometimes twice a month. Yeah, or once a month or sometimes even one year”* (K2M3)

*“We like both fish and meat, but meat is very expensive.”* (B2G4)

It became evident that fish is a more affordable animal source food in The Gambia. Serving as a primary source of animal protein, fish is widely consumed due to the country's abundant access to both salt and fresh waters ^21^. This was reflected in this study, where fish was reported as being available and commonly eaten by families. Mothers and grandmothers in both settings agreed that due to its lower cost.

*“To have fish is easier than meat.”* (K2G6).

*“…Meat is very difficult to have. And fish...is very easy to have here.”* (K3M1)

*“You only get to enjoy meat in the house if you can afford it. Fish is much cheaper”* (B2G4)

Fish consumption is recommended by WHO for children due to its high nutrient content and soft texture ^2^ and could be encouraged to be used as an animal source complementary food in The Gambia due to its higher availability and affordability when compared to meat. Dried fish, a preservation technique, is commonly consumed in The Gambia ^48^ and could be explored to settings further from the sea or with lack of refrigeration facilities.

In both settings, the high costs of animal source foods was commonly reported as a barrier to including them in children’s diets, this is reflected in other studies ^20, 23, 26^, suggesting that alongside improved availability, more affordable animal source foods would be likely to encourage consumption.

***Access to diverse foods***

In addition to availability and cost of foods, access to diverse foods impacts complementary feeding practices ^12^. In Keneba, mother’s spoke of being able to buy some locally produced foods.

*“Vegetables, rice, cous, cassava, sweet potato, maize. Everything you see in the market they are growing.”* (K2M2)

Animal source foods were less accessible, with meat being sold once a week.

*“Here, we have it (meat) here. There is a man selling here every Thursday.”* (K2M4)

The larger markets in Brikama were mentioned in both settings as a source of more diverse foods. In Keneba, Grandmothers highlighted the need to attend these markets to access foods if they *‘are not available in the village’* (K1G2). They told of seeking help from MRCG staff or friends to access these markets by public transport.

*“If they cannot buy them, they will send someone to the coast to buy them.”* (K1G1)

In Brikama, participants did not mention having to travel to buy food for children, and bought them at the *‘shops and markets in Brikama’* (B4M4)

Difficulties in travelling large distances to markets, as mentioned by participants in Keneba, can negatively affect the diversity of foods available to families, which in turn affects children’s diets ^46, 49^. This unevenly affects poorer families who are less likely to be able to travel to large markets and who are more likely to be larger with more dependent children, thus contributing to poorer quality diets for children ^47^. Children born in rural areas in The Gambia, such as Keneba, have been found to be less likely to reach minimum dietary diversity ^50^, where poverty remains a continuous challenge with approximately 69.5% of the population living below the poverty line ^51^. It was apparent that access to diverse foods was a greater barrier to mothers and grandmothers living in Keneba than Brikama.

### Strengths and limitations

The qualitative design of this study is a notable strength, allowing for a comprehensive exploration of perceptions on animal source complementary foods in this context, which is under-researched and challenging for quantitative methods alone. Despite efforts to mitigate bias, the lead investigator's lack of fluency in the local language may have introduced biases. However, using a local translator aided in understanding the regions. Conducting additional focus group discussions with other stakeholders could offer deeper insights into factors influencing complementary feeding practices. Despite limitations, these findings pave the way for future research avenues.

## Conclusion and recommendations

This study found discrepancies between current complementary feeding practices and guidelines. While knowledge on exclusive breastfeeding was in line with current recommendations, gaps existed in understanding the appropriate introduction of animal source foods. Various context-specific factors influenced the selection of diverse foods for complementary feeding. Despite some nuance between the two settings, the accessibility and affordability of animal source foods, availability and quality of IYCF services and the caregivers' knowledge and beliefs surrounding feeding practices were all key determinants of the children’s diets. Understanding these factors is crucial for improving complementary feeding practices.

Previous studies promoting awareness about animal source foods have demonstrated positive effects on children's consumption ^36, 41^. Educating caregivers about the appropriate introduction and preparation of animal source foods could yield positive outcomes in these settings. The established relationship between the MRCG and Keneba presents a unique opportunity for far-reaching complementary feeding education programmes. Past successes, such as the supplement centre's efforts, were evident but impacted by its closure, leading to reduced education on complementary feeding. In Brikama, this relationship between the MRCG and caregivers does not exist, therefore alternative methods of information dissemination should be considered. It should also be acknowledged that where these foods are not affordable or available, education alone may have limited impact. Before implementing widespread programmes to increase animal source food intake among children aged six to 24 months, comprehensive ethnographic research is recommended to observe feeding practices beyond self-reported accounts

## Ethics and consent

This study was conducted according to the guidelines laid down in the Declaration of Helsinki and all procedures involving research study participants were approved by the Medical Research Council The Gambia Scientific Coordinating Committee (LEO8788; 24 May 2023), The Gambian Ethics Committee (LEO8788; 19 June 2023), and the LSHTM Ethics Committee, (LEO28788; 28 June 2023).

Following sensitisation, informed consent was obtained from all those willing to participate. Literate participants were given the written Participant Information Sheet and signed the Informed Consent form. Illiterate participants had the full Participant Information Sheet read to them in Mandinka and used their thumbprint to sign the Informed Consent Form. All questions that arose were answered by the team. Participation was entirely voluntary, and no individuals who are not able to give consent were enrolled. An example of the Participant Information Sheet and Informed Consent is available upon request. 

## Data Availability

Further use of this data is inhibited for Ethical reasons. Any request for use of study data must be approved by the Medical Research Council Unit The Gambia (MRCG) Scientific Coordinating Committee, the Gambian Government Ethics Committee and the LSHTM Ethics Committee. All data will be in an anonymous format for external users. Data sharing will agree with the MRCG policy on research data sharing. All reasonable requests will be granted. For assistance please contact the corresponding author ( ccerami@mrc.gm). *Figshare:* [Extended Data: Exploring mothers' and grandmothers' perceptions of animal-source complementary foods in the diets of young children in The Gambia: A qualitative study]. https://doi.org/10.6084/m9.figshare.28256009.v1 ^52^. *This project contains the following extended data: * (1) Focus Group Discussion Guide. (2) Demographic questionnaire. (3) Participant information sheet (4) Informed Consent Form. Data are available under the terms of the Creative Commons Attribution 4.0 International license (CC-BY 4.0).
