# Supplementary material for: Exploring mothers' and grandmothers' perceptions of animal-source complementary foods in the diets of young children in The Gambia: A qualitative study
Source: Wellcome Open Res. 2025 Jul 23;10:65. Originally published 2025 Feb 12. [Version 2] doi: 10.12688/wellcomeopenres.23090.2 (PMC12423624; doi:10.12688/wellcomeopenres.23090.2)
Supplement: Peer review history

# Reviewer report: Reviewer response for version 2

**DOI:** 10.21956/wellcomeopenres.27171.r127676

**Published:** 2025-09-10

**Authors:** Kulnipa Kittisakmontri

**Affiliations:** 1 Pediatrics, Chiang Mai University Faculty of Medicine, Chiang Mai, Chiang Mai, Thailand; 2 University College London Great Ormond Street Institute of Child Health, London, UK

The authors have well addressed my comments. The revised version was more explicit and satisfied.. I spotted a repeated sentence " ***“For the kids, they can eat banana and eggs. It doesn't affect them.*” *(B5G4)" * **please check.

Is the work clearly and accurately presented and does it cite the current literature?

Yes

If applicable, is the statistical analysis and its interpretation appropriate?

Not applicable

Are all the source data underlying the results available to ensure full reproducibility?

No source data required

Is the study design appropriate and is the work technically sound?

Yes

Are the conclusions drawn adequately supported by the results?

Partly

Are sufficient details of methods and analysis provided to allow replication by others?

Yes

Reviewer Expertise:

Pediatric nutrition specialist

I confirm that I have read this submission and believe that I have an appropriate level of expertise to confirm that it is of an acceptable scientific standard.

# Reviewer report: Reviewer response for version 2

**DOI:** 10.21956/wellcomeopenres.27171.r127677

**Published:** 2025-07-28

**Authors:** Aaron Kobina Christian

**Affiliations:** 1 Regional Institute for Population Studies, University of Ghana, Legon-Accra, Accra, Ghana

Thank you. The Authors have addressed the comments raised satisfactorily. I recommend the paper indexed

Is the work clearly and accurately presented and does it cite the current literature?

Partly

If applicable, is the statistical analysis and its interpretation appropriate?

Not applicable

Are all the source data underlying the results available to ensure full reproducibility?

Partly

Is the study design appropriate and is the work technically sound?

Yes

Are the conclusions drawn adequately supported by the results?

Yes

Are sufficient details of methods and analysis provided to allow replication by others?

Yes

Reviewer Expertise:

Population Health and Nutrition

I confirm that I have read this submission and believe that I have an appropriate level of expertise to confirm that it is of an acceptable scientific standard.

# Reviewer report: Reviewer response for version 1

**DOI:** 10.21956/wellcomeopenres.25426.r120338

**Published:** 2025-03-20

**Authors:** Aaron Kobina Christian

**Affiliations:** 1 Regional Institute for Population Studies, University of Ghana, Legon-Accra, Accra, Ghana

**The authors need to be clear or answer the following:**

 1. While I understand that they said there was no exclusion criteria, does it mean they interviewed even women with no experience of caring for children? 

 2. Can the authors briefly discuss the framework method "as outlined by Gale et al."

 3. The author indicate that "generally the awareness of foods suitable for children aligned with recommendation of WHO" without specifying that with any quotes. 

 4. The quote provided concerning taboos speaks to mothers own feeding restrictions not feeding their children. Can authors provide a better quote that speaks about "feeding their children"

Is the work clearly and accurately presented and does it cite the current literature?

Partly

If applicable, is the statistical analysis and its interpretation appropriate?

Not applicable

Are all the source data underlying the results available to ensure full reproducibility?

Partly

Is the study design appropriate and is the work technically sound?

Yes

Are the conclusions drawn adequately supported by the results?

Yes

Are sufficient details of methods and analysis provided to allow replication by others?

Yes

Reviewer Expertise:

Population Health and Nutrition

I confirm that I have read this submission and believe that I have an appropriate level of expertise to confirm that it is of an acceptable scientific standard, however I have significant reservations, as outlined above.

# Reviewer report: Reviewer response for version 1

**DOI:** 10.21956/wellcomeopenres.25426.r119707

**Published:** 2025-03-19

**Authors:** Kulnipa Kittisakmontri

**Affiliations:** 1 Pediatrics, Chiang Mai University Faculty of Medicine, Chiang Mai, Chiang Mai, Thailand; 2 University College London Great Ormond Street Institute of Child Health, London, UK

The manuscript was well written and the concept of this study was interested.

**I have few comments as follows:**

**Theme 1**

**Caregiver knowledge** - It's difficult to conclude that there was a knowledge gap between mothers and grandmothers because there was only one topic, age of solid food introduction, presented in this subtitle. I suggested to add 2 or more topics to demonstrate a knowledge gap among them. Did the grandmothers had more false belief in animal source food than mothers?

**Family foods** - it was not clear whether the family foods contained animal source foods or not. Did they consume any meat or products of  their own livestock? I understand that some villagers just rear the livestock for only financial purpose. If the authors aimed to show influences of family foods on child's diet, what food they consumed on daily basis should clearly described.

Is the work clearly and accurately presented and does it cite the current literature?

Yes

If applicable, is the statistical analysis and its interpretation appropriate?

Not applicable

Are all the source data underlying the results available to ensure full reproducibility?

No source data required

Is the study design appropriate and is the work technically sound?

Yes

Are the conclusions drawn adequately supported by the results?

Partly

Are sufficient details of methods and analysis provided to allow replication by others?

Yes

Reviewer Expertise:

Pediatric nutrition specialist

I confirm that I have read this submission and believe that I have an appropriate level of expertise to confirm that it is of an acceptable scientific standard, however I have significant reservations, as outlined above.

# Response

**Published:** 2025-07-09

**Authors:** Carla Cerami

**Affiliations:** London School of Hygiene & Tropical Medicine, The Gambia

Thank you for your comments on the manuscript, it has been useful to be able to clarify the points that you have raised.  

 Q1. We agree that the conclusion regarding the knowledge gap could be more clearly articulated. In response, we have revised the manuscript to more explicitly state that the knowledge gap was primarily on the age of complementary food introduction (see page 11). While we’re aware that this represents just one aspect of infant and young child feeding (IYCF) practices, we believe it is important to highlight, given its significance.  

 Q2. We have revised the manuscript to more clearly describe the animal-source foods included in family diets. Specifically, we have added contextual detail and a supporting quote to clarify this point. This additional information helps to better illustrate the influence of family foods on children’s diets, thank you for raising it. (See revised section on page 12.)
